# Nanomedicine: New Frontiers in Fighting Microbial Infections

**DOI:** 10.3390/nano13030483

**Published:** 2023-01-25

**Authors:** Mohammad Reza Mehrabi, Madjid Soltani, Mohsen Chiani, Kaamran Raahemifar, Ali Farhangi

**Affiliations:** 1 Department of NanoBiotechnology, Pasteur Institute of Iran, Tehran 13169-43551, Iran; 2 Department of Mechanical Engineering, K. N. Toosi University of Technology, Tehran 19967-15433, Iran; 3 Advanced Bioengineering Initiative Center, Multidisciplinary International Complex, K. N. Toosi University of Technology, Tehran 14176-14411, Iran; 4 Centre for Biotechnology and Bioengineering (CBB), University of Waterloo, Waterloo, ON N2L 3G1, Canada; 5 Department of Electrical and Computer Engineering, University of Waterloo, Waterloo, ON N2L 3G1, Canada; 6 Data Science and Artificial Intelligence Program, College of Information Sciences and Technology (IST), Penn State University, State College, PA 16801, USA; 7 Department of Chemical Engineering, University of Waterloo, 200 University Avenue West, Waterloo, ON N2L 3G1, Canada; 8 School of Optometry and Vision Science, Faculty of Science, University of Waterloo, 200 University Avenue West, Waterloo, ON N2L 3G1, Canada

**Keywords:** microbial infection, nanomedicine, vaccine, diagnosis, therapy

## Abstract

Microbes have dominated life on Earth for the past two billion years, despite facing a variety of obstacles. In the 20th century, antibiotics and immunizations brought about these changes. Since then, microorganisms have acquired resistance, and various infectious diseases have been able to avoid being treated with traditionally developed vaccines. Antibiotic resistance and pathogenicity have surpassed antibiotic discovery in terms of importance over the course of the past few decades. These shifts have resulted in tremendous economic and health repercussions across the board for all socioeconomic levels; thus, we require ground-breaking innovations to effectively manage microbial infections and to provide long-term solutions. The pharmaceutical and biotechnology sectors have been radically altered as a result of nanomedicine, and this trend is now spreading to the antibacterial research community. Here, we examine the role that nanomedicine plays in the prevention of microbial infections, including topics such as diagnosis, antimicrobial therapy, pharmaceutical administration, and immunizations, as well as the opportunities and challenges that lie ahead.

## 1. Introduction

Antibiotics and vaccines are among the greatest medical advances. Over the previous century, broad-spectrum medicines and vaccinations greatly lowered infectious disease morbidity and mortality [1,2,3]. Infectious disease mortality in the US declined dramatically from 797 to 59 deaths per 100,000 between 1900 and 1996, with the lowest rate of 36 fatalities per 100,000 in 1980. In recent decades, some worrying patterns have evolved that jeopardize such progress. According to the World Health Organization’s Global Health Study from 2016, infectious and parasitic diseases are responsible for 9.7 percent of global deaths. The top five causes of death worldwide are as follows: TB (2.3%), diarrheal bacterial infections (2%), meningitis (0.5%), bacterial sexually transmitted disorders (syphilis, chlamydia, and gonorrhea, 0.2%), and encephalitis (0.2%) [4]. The Global Burden of Diseases consortium reports that Shigella and enterotoxigenic Escherichia coli are the most common and lethal bacteria that cause infectious diarrhea [5,6]. In 2016, infectious diarrhea was the eighth leading cause of death across all ages and the fifth leading cause of death among children. Pneumococcus is the largest cause of years of disability across the globe, due to an increase of 2.82 million cases of meningitis in 2016 [7]. About 11 million people died from sepsis-related causes in 2017 [8]. These numbers are significantly higher than the global average in impoverished nations because of the lack of universal health systems, public health issues, potable drinking water, and financial resources [4,6]. Antibiotic overuse has been linked to its emergence. Antibiotic ineffectiveness that is caused by rising drug resistance is a major threat to public health. Some researchers have even predicted that the 21st century will be the “postantibiotic era” [9,10]. Multidrug resistance (MDR) is a phenomenon that can occur in some bacteria [11]. Some multidrug-resistant infections are resistant to conventional therapies. An alarming example of multidrug resistance is the increasing number of strains of methicillin-resistant Staphylococcus aureus (MRSA) that are also resistant to vancomycin (VRSA), complicating therapy because vancomycin is usually the last line of defense against S. aureus infections [12]. Medication resistance, and new antimicrobial drugs, are falling behind the rapid pace at which microbes evolve [13]. On the other hand, traditional vaccinations that use live attenuated microorganisms, killed microbes, or microbial components, have proven to be crucial to infectious disease control, although some do not protect well. In addition, immunocompromised people should not utilize some live vaccines. No vaccinations are available for many infectious illnesses. In order to overcome these issues, a variety of vaccines that are based on isolated proteins, polysaccharides, or naked DNA encoding a protective antigen, are being produced. Although these can be safer, more defined, and less reactogenic than many vaccinations, they are often poor immunogens that need adjuvants to improve their activity. The pharmaceutical industry has slowed down the development of novel antibiotics, especially for MDR Gram-negative superbugs, due to low returns on investment and R&D objectives [14,15].

**Figure 1:**
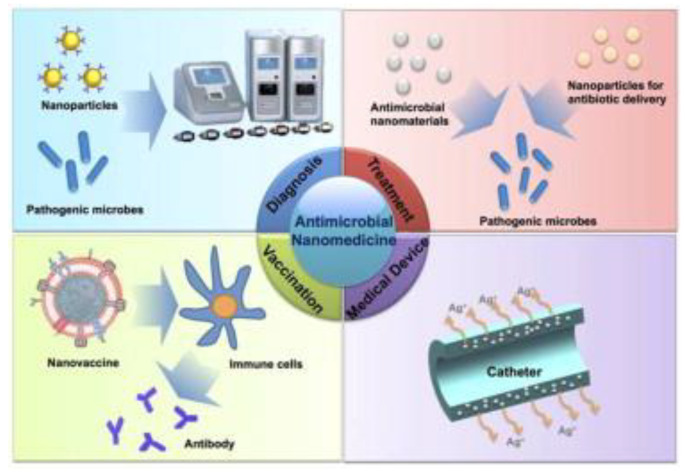
Applications of nanomedicine in the treatment of infectious diseases caused by microbes. Reproduced with permission from [24]. Copyright Elsevier, 2014.

The pharmaceutical and biotechnology industries have been revolutionized by nanomedicine, or the application of nanotechnologies in medicine [16,17,18,19,20,21,22]. Clinical use approval has been granted for close to one hundred different nanomedicine products as of 2020. These products range from medication delivery and imaging to implantable biomaterials and medical devices [18]. Nanotechnologies can also tackle nearly every element of microbial illness (Figure 1). Nanomaterials’ unique physicochemical properties have helped to detect microbial diseases quickly, sensitively, and selectively. In addition, several inorganic and organic nanoparticles have significant intrinsic antibacterial capabilities that are rarely manifested in bulk form. More importantly, certain nanomaterials can reduce antibiotic resistance by weakening the resistance pathways. In addition, nanoparticles for antimicrobial drug delivery overcome resistance and have fewer adverse effects than the conventional antibiotics. Medical equipment can also inhibit bacteria adherence and infection by using antimicrobial nanoparticles. Last but not least, nanomaterials can boost immune responses to microbial illness as vaccine adjuvants or delivery vehicles. For antigens that would otherwise disintegrate quickly after injection, or cause a transient, the localized immune response can be delivered in a more stable form via encapsulation in nanoparticles. The possibility of integrating multiple antigens onto a single particle in order to protect against more than one illness is also being investigated, as is the use of nanoparticles to deliver vaccines by non-traditional routes, such as topical, inhalational, or optical delivery [23]. Here, we focus on the recent developments in nanotechnology that have been applied to the fight against infectious microbes.

## 2. Vaccination

It has been demonstrated that utilizing the host’s immune system to recognize and kill germs protects the host against microbial infection. Pathogen-associated molecular patterns help the innate immune system to identify pathogens that breach the host’s physical barriers [25]. Antigen-specific adaptive immune responses against bacterial infections can persist for decades after activating antigen-presenting cells (APCs) [26]. The protective response may delay bacteremia and septic shock, giving antibiotics more time to work. Microbe vaccines vary in immunogenicity and safety. Live attenuated bacterial vaccines raise concerns about pathogenicity reversion, vector immunity, and immune-compromised safety [27,28].

**Figure 2:**
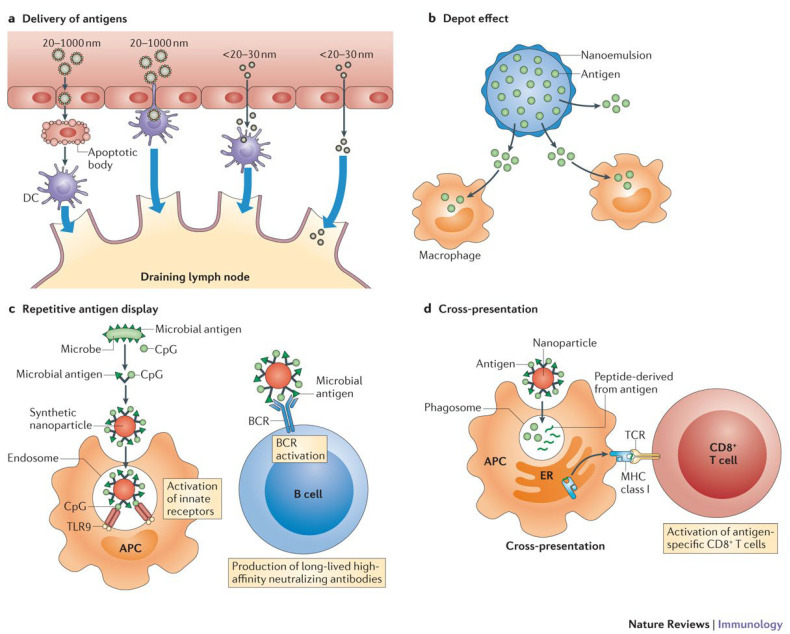
Immune response induction and how nanoparticles affect it [30]. Reproduced with permission from [30]. Copyright Springer, Nature, 2013.

Isolated proteins, polysaccharides, and bare DNA are used to create next-generation bacterial vaccines, thanks to biotechnology [29]. Compared to vaccinations that are made from live, attenuated microbes, novel vaccines have a lower immune response. One possible answer lies in the use of nanotechnology to increase the effectiveness of vaccines on the immune system. Nanoparticle antigens elicit systemic and local humoral immune responses, including IgG and IgA antibodies and cellular responses from Th1, Th2, and Th17 cells [30]. Increased tissue penetration, access to the lymphatics, and preferential uptake by APCs are just a few examples of how nanoparticles can stimulate the immune system (Figure 2). Another way in which nanoparticles can do this includes the depot effect, which stabilizes the antigens and controls their sustained release. The depot effect involves the antigen and the adjuvant being displayed on the particle surface repeatedly in order to stimulate B cell receptor co-aggregation, triggering, and activation. Nanoparticle delivery technologies act as their adjuvants [30,31].

Nanoparticles deliver mucosal vaccinations well. Mucosal surfaces contain nearly 80% of immunocytes and are the first line of defense [32]. A total of 70% of pathogens enter the body through the mucosal surfaces [33]. Thus, a long-term mucosal immune response protects the host from bacterial infection. Mucosal vaccination induces mucosal and systemic immunity, while subcutaneous or intramuscular vaccines only induce a weak mucosal immune response [34]. Thus, intranasal, inhalational, and gastrointestinal mucosal vaccinations are becoming popular. Since the antigen must pass through several barriers before reaching the APCs, mucosal immunization is limited. Mucosal vaccination could benefit from immunostimulatory nanoparticle delivery vehicles [34]. The main sites of mucosal immunological activation are located in organized mucosa-associated lymphoid tissue (MALT), which can be reached by these nanoparticles. Antigen-loaded nanoparticles that are engineered with UEA-1 lectin, which selectively binds to M cells in MALT, have led to a two- to four-fold rise in antibody titers [35].

### 2.1. Adjuvant

Effective non-inflammatory mucosal adjuvants include nanoemulsions, which are oil-in-water emulsions containing droplets on the nanoscale [36]. Potentially enhanced antigen absorption, monocytes, and granulocyte recruitment, and cytokine and chemokine release, may result from nanoemulsion adjuvanticity [30]. After one or two mucosal injections, serum IgG and bronchial IgA and IgG antibodies were generated in mice and guinea pigs by recombinant anthrax protective antigens that were combined in nanoemulsion [37]. The commercial human anthrax vaccine schedule consists of six subcutaneous injections that are given at 18-month intervals, followed by annual booster shots. In order to boost immunity against Burkholderia, scientists used nanoemulsion as a novel mucosal adjuvant for the intranasal injection of Burkholderia multivorans outer membrane proteins antigen in vaccinated mice. Neutralizing activity against Burkholderia was demonstrated by these immune responses [38].

Cationic liposomes are used as an adjuvant in vaccinations. A cationic liposome-based adjuvant called CAF01 has been proven to improve vaccine-candidate immune responses and is currently in clinical testing [39]. In a study that was aimed at creating more effective and safer tuberculosis vaccines, researchers found that combining CAF01 with a synthetic mycobacterial glycolipid induced significant and protective Th1 and Th17 responses [40]. DC absorption and activation were prolonged by CAF01. The adjuvants for parenteral and mucosal vaccines were cationic liposomes containing non-coding plasmid DNA. Mice of the BALB/c strain were completely protected from a normally deadly lung challenge when they were given a liposome–DNA complex as a mucosal adjuvant along with heat-killed *Burkholderia pseudomallei* (*B. pseudomallei*) [41].

### 2.2. Vaccine Delivery

Small molecules, peptides, proteins, and nucleic acids can all be carried by polymeric nanoparticles. Antigens and adjuvants can be transported through synthetic polymers, which can then be injected into a patient [42]. An increase in CD4+ and CD8+ T cell subsets, and Th1 antibody titers that were 64-fold higher than Th2, were observed after exposure to PLGA nanoparticles expressing a recombinant major outer membrane protein of Chlamydia trachomatis (*C. trachomatis*) [43]. Inactivated bacterial toxoid vaccinations have been widely utilized to prevent and cure microbial illnesses by promoting antitoxin immunity. Eliminating toxin virulence while maintaining antigenicity is still difficult. Zhang and colleagues used erythrocyte membrane-coated polymeric nanoparticles to securely administer non-disrupted pore-forming toxins for immune processing (Figure 2) [44]. The nanoparticle-detained toxin gave mice a greater protection against toxin-mediated deleterious effects, neutralized poisons, and 100% survival. Chitosan and pullulan have been used to provide antigens against *C. trachomatis* and *Streptococcus pneumonia* [45,46]. Chitosan promoted cytokine synthesis, making it an adjuvant. Chitosan-modified antigen-loaded poly(e-caprolactone) nanoparticles increased IgG and IgA antibody responses [47].

In the case of protein oligomerization, self-assembling peptide nanoparticles (SAPNs) take the form of icosahedral symmetric assemblies. These aggregates are called “virus-like particles” (VLPs) due to their superficial similarity to viral capsids. SAPNs serve as a framework that allows for the highly exposed presentation of inserted protein epitopes or domains [48]. The introduction of different antigens into SAPNs can stimulate the production of antibodies against low-immunogenic antigens. In the absence of an adjuvant, animals that are immunized with SAPNs paired with an immunodominant B cell epitope that is derived from the circumsporozoite protein of Plasmodium berghei developed high-affinity, long-lasting T cell-dependent antibodies [49].

ISCOMs, or immune-stimulating complexes, are cage-like antigen delivery vehicles that are composed of cholesterol, phospholipid, and saponin [50]. ISCOMs have the potential to activate the IL-12-dependent components of the innate immune system and induce MHC class I and class II antigen presentation. ISCOMs have also demonstrated effectiveness as mucosal vaccines, especially when they are administered intranasally [51]. ISCOMs stimulate protective immune responses against *Helicobacter pylori*, *Anaplasma marginale*, *Mycoplasma mycoides*, *Mycobacterium tuberculosis*, *Corynebacterium diphtheriae*, *Streptococcus pyogenes*, *Moraxella Bovis*, and *Chlamydia trachomatis* [50].

## 3. Diagnosis

Contagious bacteria can spread infectious illnesses from sick people to healthy people. Thus, rapid, sensitive, and specific pathogen detection is essential for detecting infection sources, treating patients, and preventing illness [52,53]. Some of these illnesses are difficult to diagnose due to the complexity and diversity of the microorganisms and the long incubation period before the clinical symptoms arise (from minutes to years). ELISA and PCR are sensitive and reproducible molecular methods for microbial infection detection. However, these methods involve tedious sample preparation and extensive readout periods, which may delay time-critical infection detection and treatment, such as bacterial sepsis. These detection methods are also difficult to use in underdeveloped nations and rural parts of industrialized countries, where microbial infectious illnesses are more common.

Nanotechnology can produce rapid, sensitive, specific, and cost-effective microbial illness diagnosis methods [54]. Detecting target molecules/microbes in a complex sample matrix requires selective capture and separation. Nanotechnology can aid both of these processes, and nanoparticles’ unique physicochemical features may allow the recording of a single binding event. Nanoparticles containing affinity probes, such as antibodies and nucleic acids, can label or capture the targets by recognizing microbial biomarkers. Nanoscale ligand arrays that target specific pathogens and surface patterning could also significantly improve the detection of infectious diseases. Nanoparticles that are made of magnetic materials, gold (Au), and fluorescent dyes are used in microbiological diagnosis.

### 3.1. Magnetic Nanoparticles

**Figure 3:**
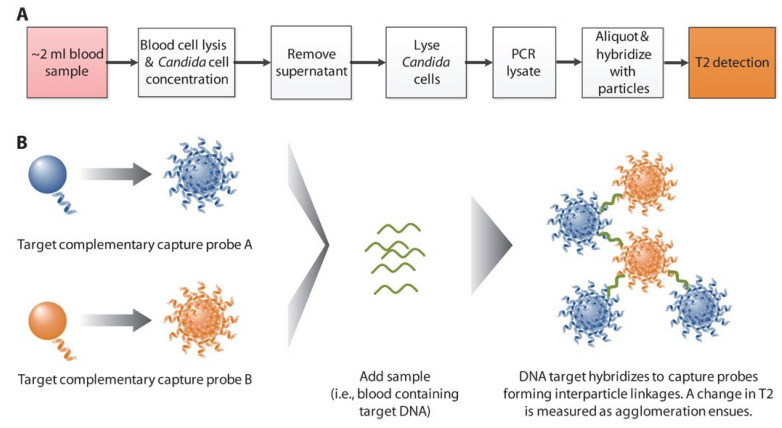
(**A**) Candida T2MR assay process. (**B**) T2MR detecting particle reagent schematic. SPIONs covalently conjugate oligonucleotide probes. Each target had two nanoparticle populations with a target-complementary probe. These nanoparticles aggregate when hybridized to the target strand amplified in excess by asymmetric PCR, changing the sample’s T2MR signal. DNA concentration increases clustering. Reproduced with permission from [59]. Copyright Elsevier, 2017.

Superparamagnetic iron oxide nanoparticles (SPIONs) have been the subject of many studies as contrast agents for magnetic resonance imaging (MRI) [55,56,57]. Research on the use of magnetic nanoparticles that are coated with a probe in microbiological diagnostics has also progressed significantly in recent years. Lowery and coworkers created a SPION diagnostic technique based on T2-magnetic resonance (T2MR) that can detect five Candida species in whole blood samples in a fast manner and with high reproducibility within three hours [58]. The T2MR signal is significantly altered when oligonucleotide-decorated SPIONs hybridize with amplified Candida DNA (Figure 3). Based on this method, T2Candida is currently being utilized in clinical studies. Magneto-DNA nanoparticles were produced by Weissleder and colleagues for clinical pathogen profiling [24]. These nanoparticles target bacterial ribosomal RNA. Using a tiny nuclear magnetic resonance (NMR) device, the assay was able to detect and phenotype 13 different bacterial species that were present in the clinical specimens in under two hours.

Magnetic nanoparticles can be used to enrich, wash, and resuspend targets from a complex biological matrix with the help of magnetic fields that can be controlled. It is possible to identify bacteria in a sensitive and multiplex manner using this magnetic nanoparticle profile and new detection technologies. Matrix-assisted laser desorption/mass spectrometry, which is also known as MALDI-MS, is a technique that has been used to rapidly and accurately identify bacteria [60]. This technique is based on the mass spectrometry properties of common bacterial species. Rapid bacterial screening in clinical samples, such as whole blood, is made possible through magnetic nanoparticle-based sample preparation and concentration, as well as MALDI-MS detection [60,61]. In addition, ligand-modified magnetic nanoparticles and magnetic microfluidic devices can eliminate pathogens and endotoxins from the bloodstream [62,63]. When they are added to bovine whole blood, magnetic nanoparticles that have been coated with the synthetic ligand bis-Zn-DPA have the potential to eliminate *E. coli* with a clearance rate of around 100% at 60 mL/h.

In order to assess the metabolic activity and antibiotic resistance in bacteria, magnetic nanoparticles were used to track nutrient consumption (e.g., starch). In order to determine the susceptibility of bacteria in blood to antibiotics, Perez and colleagues [63] devised two methods based on SPION that make use of magnetic relaxation. Low metabolic activity or bacterial growth rates can trigger the assembly of Con A-conjugated SPIONs or dextran-coated SPIONs supplied with free Con A, resulting in a shift in T2MR. After 2.5 h, or 5 min, depending on whether or not free Con A is present, ampicillin susceptibility can be determined using a dextran-coated SPION competition assay. There is no need to incubate the sample cells for 24 h using this method, yet it provides just as precise an assessment of antibiotic sensitivity as the turbidity method.

### 3.2. Au Nanoparticles

Au nanoparticles’ unique optical and electrochemical characteristics, and their ability to be surface-functionalized with probes, have made them popular sensing materials [64]. Since Mirkin and colleagues’ pioneering work [65], oligonucleotide-functionalized Au nanoparticles have been frequently utilized as probes to quickly identify viruses whose genome sequences include distinctive nucleic acid fingerprints. Oligonucleotide–Au nanoparticles that are hybridized with target nucleic acids create a polymeric network and move the plasmon resonance peak [65]. Storhoff and colleagues devised a “spot-and-read” colorimetric approach for recognizing MRSA strains’ mecA genes using Au nanoparticles’ distance-dependent optical characteristics [66]. When they were spotted over an illuminated glass waveguide, these nanoparticles hybridized and changed color, detecting the nucleic acids with zeptomole sensitivity.

**Figure 4:**
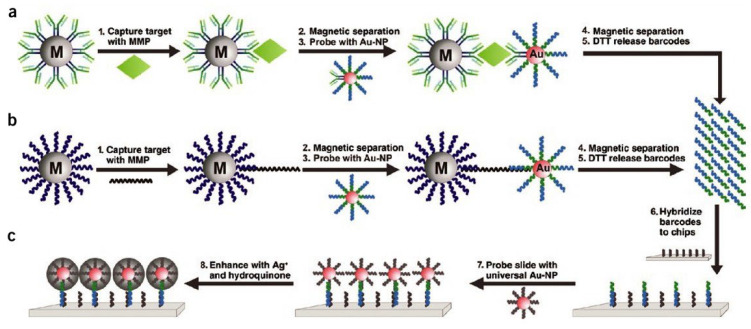
Assay using bio-barcodes for the detection of DNA and proteins. A representation in schematic form of (**a**) the identification of proteins by the use of the bio-barcode test; (**b**) detection of nucleic acids by the use of the bio-barcode test; as well as (**c**) the econometric detection method. Reproduced with permission from [24]. Copyright Elsevier, 2014.

Au nanoparticle probes that are tagged with oligonucleotides and Raman-active dyes can be used for the multiplexed detection of oligonucleotide targets with good sensitivity and selectivity [67]. At 20 femtomolar concentrations, six distinct DNA targets were distinguished by Au nanoparticle probes that were tagged with Raman rays. Using this detection strategy, Mirkin and coworkers created a bio-barcode test for ultrasensitive nucleic acid and protein targets [68]. For magnetic separation and dithiothreitol (DTT)-mediated release of barcode strands, as shown in Figure 4, the targets of interest are sandwiched between Au nanoparticles and magnetic microparticles. The Verigene test, which was developed by Nanosphere, Inc., detects Gram-positive and Gram-negative bacteria directly from blood samples using in vitro methods. After a positive blood culture, the results can be delivered in 2–2.5 h with this test, compared to the normal 2–4 days with traditional microbiological procedures. This test is two- to three-orders-of-magnitude more sensitive than ELISA-based approaches [69].

Affinity probes besides oligonucleotides have been described and demonstrated to be useful for tagging Au nanoparticles for bacterial diagnosis. Gold nanoclusters that were enclosed in lysozymes and designed to interact with peptidoglycans on bacterial cell walls were produced to concentrate pathogenic germs for MALDIMS-based identification [70]. Stabilized gold nanoclusters against S. aureus and MRSA via human serum albumin or its binding peptide motif were produced [71]. Gold nanoparticle antimicrobial resistance can also be measured by monitoring the surface plasmon band shifts that are produced by Con A-induced clustering of extra-coated Au nanoparticles in a bacterial solution with starch [72].

### 3.3. Fluorescent Nanoparticles

Microbial detection has also been conducted with the use of nanomaterials or nanoparticles with fluorescent dyes. Antibody-conjugated silica nanoparticles containing hundreds of fluorescent dye molecules for signal amplification were produced by Tan and colleagues to allow for the in situ detection of single bacterial cells in less than twenty minutes [73]. Multicolored FRET silica nanoparticles were created by co-encapsulating three tandem dyes that emit various hues when they are excited with a single wavelength [74]. Different monoclonal antibody-conjugated FRET silica nanoparticles detected various bacterial targets simultaneously. Quantum dots (QDs), which are fluorescent semiconductor nanoparticles, have several advantages over traditional fluorophores, including photobleaching resistance and size-tunable wide absorption spectra with narrow emission [75]. QDs’ optical properties and variable surface chemistry make them a promising medium for complicated sample analysis and Listeria monocytogenes detection [76]. These affinity probes are promising for the high-throughput microbial identification of biological and environmental samples due to their chemical and physical plasticity and unique interactions with molecular targets or pathogens. Miniaturized devices with reduced sample quantities, quicker readouts, and improved sensitivity and accuracy will be created. Most nanoparticle-based diagnostic techniques use targeted probes to recognize known bacterial genome sequences/biomarkers and may not detect altered or novel bacteria strains. As drug-resistant strains grow, diagnostic nanotechnology that can detect germs and determine their sensitivity to antimicrobials is another key avenue.

## 4. Treatment

Antibiotic resistance is on the rise, posing a risk to the general population. Mutation and horizontal gene transfer are two mechanisms by which bacteria acquire resistance [77]. Reduced drug uptake and drug efflux from the microbial cell, the increased synthesis of a competitive inhibitor of antibiotics, and changes in the antibiotic-binding substrate are the root causes of antimicrobial drug resistance [78]. Chronic infections that are induced by biofilms and intracellular bacteria, including Mycobacterium leprae, Chlamydia, Listeria, and others, are another major obstacle in antimicrobial therapy [79,80]. Biofilm is an extracellular polymeric material (EPS) matrix that surrounds bacterial cells [81,82]. It traps and degrades antibiotic compounds, preventing diffusion. Biofilm bacteria can withstand various antibiotics 1000 times better than planktonic bacteria [83]. The host cell protects the intracellular bacteria from several drugs. Chronic infections require frequent high-dose antibiotics, therefore, their eradication is challenging.

**Figure 5:**
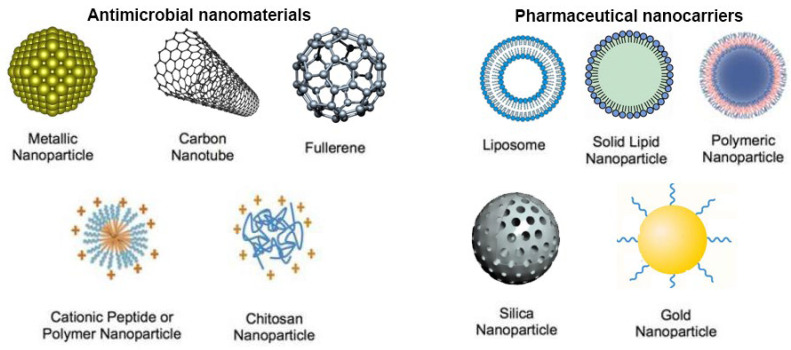
Antimicrobial nanomaterials and nanoparticle-based drug delivery systems: A schematic overview.

Nanomedicine can cure microbial resistance without promoting it. Antimicrobial nanomaterials targeting numerous routes and the nanoparticle-based delivery of antibiotics might achieve this. Antimicrobial nanotherapeutics that suppress biofilms and target intracellular microorganisms may cure persistent infections. Nanomedicine is used to generate inorganic and organic nanomaterials with intrinsic antibacterial characteristics (Figure 5A) and nanoparticle-based antimicrobial medication delivery (Figure 5B).

### 4.1. Antimicrobial Nanomaterials

#### 4.1.1. Inorganic Nanoparticles

Metals and metal oxides: For centuries, metals and metal oxides have been used as bactericidal agents in infection control [84,85,86]. Photocatalysis, photothermal effects, and ROS-stimulating activities are unique to metal and metal oxide nanoparticles [87,88]. These nanoparticles’ huge surface-area-to-volume ratio allows easy surface functionalization for more potent antibacterial agents.

Metal nanoparticles that are made of silver (Ag) have been studied the most extensively. Several drug-resistant organisms, including Pseudomonas aeruginosa, ampicillin-resistant Escherichia coli O157:H7, and erythromycin-resistant *Streptococcus pyogenes*, may be susceptible to their toxicity [89]. The effects of Ag on bacteria and other microorganisms are largely unknown. Ag compounds may be involved in bacterial cell death by both direct and indirect interactions with membranes, DNA, enzymes, and proteins [87]. The transport of Ag+ ions, which are formed when Ag is exposed to ambient O2 and dissolved in water, is essential for Ag’s antibacterial effect. Since smaller Ag nanoparticles have a higher surface-area-to-volume ratio, their rate of Ag+ release and antibacterial activity are affected [42,90]. When compared to bulk Ag, Ag nanoparticles have significantly higher antibacterial activity. Their surface roughness, hydrophobicity, oxidation state, and functionalization also impact Ag nanoparticles’ antibacterial activities [91]. For instance, glucosamine modification of Ag nanoparticles’ surfaces improves their antibacterial effectiveness by entering both Gram-negative and Gram-positive bacterial cells [92].

Tellurium (Te) and Bismuth (Bi) have also been researched for antibacterial therapy. The nanoparticles outperformed Ag nanoparticles in antibacterial activity and lower toxicity [93]. ZnO, CuO, TiO_2_, Al_2_O_3_, and CeO_2_ nanoparticles are also antibacterial [94]. For example, ZnO nanoparticles inhibit *E. coli* O157:H7 [95]. Metal oxide nanoparticles suppress bacteria by the photocatalytic creation of ROS (which destroys their cellular components), the reduction of bacterial membrane integrity, the disruption of energy transduction and transport activities, and the reduction in respiratory enzyme activity and DNA synthesis [96].

Metal and metal oxide nanoparticles as antimicrobials are hard for microorganisms to resist. Metals/metal oxides have several mechanisms of action, making microorganism resistance unlikely, unless multiple mutations occur concurrently. Ag, Bi, ZnO, and TiO_2_ nanoparticles also inhibit biofilm [97]. Bi nanoparticles reduced *Streptococcus* mutant’s growth by 69% and biofilm formation by 100% [98]. However, metal and metal oxide nanoparticles are mostly used in medical devices to prevent bacterial adhesion and infection. Safety concerns may limit their antimicrobial therapeutic use [99]. ZnO and TiO_2_ damage DNA, and CuO nanoparticles cause oxidative lesions [99]. Repeated injections accumulated Ag nanoparticles in the liver, the lung, and the spleen, which could damage these organs [100]. These findings suggest that chronic exposure should be monitored for toxicity. Furthermore, some metal and metal oxide nanomaterials may pose additional risks. Al_2_O_3_ nanoparticles promoted the horizontal conjugative transfer of MDR genes, increasing antibiotic resistance [101].

Carbon: Although they are still under research, carbon-based nanomaterials, including SWCNTs, MWCNTs, and fullerene, have been used in antibacterial applications [102]. These nanoparticles may kill bacteria through cell membrane disruption or photothermal/photodynamic characteristics [103]. Oxidative stress affects the bacterial membrane integrity and metabolic activity, making SWCNTs effective against Gram-positive and Gram-negative bacteria [104]. Fullerene has also been shown to be highly antibacterial. Some investigations imply that the oxidative by-products from fullerene production may cause toxicity [105]. Hydrophilic fullerene derivatives produce ROS efficiently and can be employed as photosensitizers in antimicrobial photodynamic treatment (PDT). Antimicrobial PDT illuminates microbial pathogens and develops no innate resistance [106].

#### 4.1.2. Peptide- and Polymer-based Nanoparticles

Cationic peptides: Cationic antimicrobial peptides (CAPs)—nature’s antibiotics—are short amphipathic peptides that are found in all living forms, and they are effective against many microorganisms, including MDR bacteria [107]. High-multicellular organisms’ microbial defense systems include CAPs [108]. CAPs harm negatively charged microbial membranes, due to their cationic and hydrophobic characteristics. Cationic peptides’ cytotoxicity (e.g., hemolysis), enzymatic instability, and immunological surveillance restrict the antibacterial use of hundreds of CAP sequences [109]. Thus, placing CAPs on silica or paramagnetic nanoparticles protects the peptides from proteolytic breakdown and immunological recognition [110].

**Figure 6:**
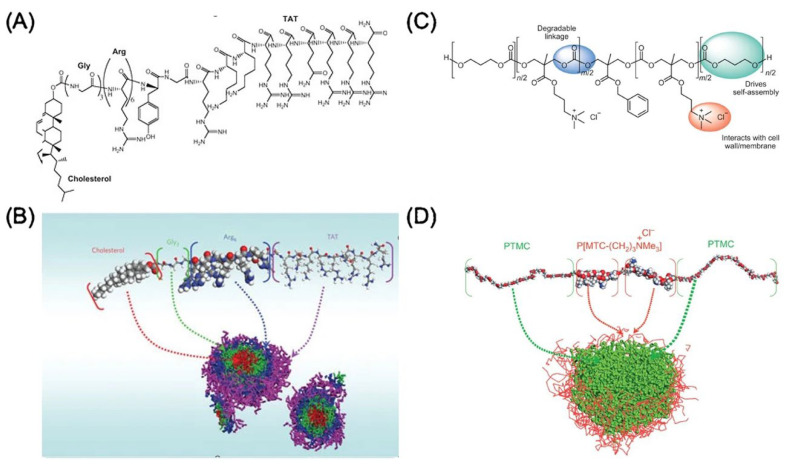
Images (**A**) and (**B**) are the chemical structure of the proposed peptide containing cholesterol, glycine, arginine, and TAT, and represent the formation of micelles. Reproduced with permission from [121], American Chemical Society, 2013. Images (**C**) and (**D**) are the chemical structure of cationic amphiphilic polycarbonate and represent the formation of micelles, as simulated by the Materials Studio program utilizing molecular modeling. Reproduced with permission from [122]. Copyright American Chemical Society, 2015.

CAPs with cationic and amphipathic characteristics can self-assemble into nanostructures that are less toxic and more effective against bacteria in vivo than unassembled peptides [111]. Furthermore, nanostructure morphology has been linked to bioactivity, suggesting that the nanostructure itself may contribute to antibacterial activity [112]. Yang and colleagues created an amphiphilic peptide with cell-penetrating peptide TAT, six arginine residues, and cholesterol that can self-assemble into core–shell nanoparticles (Figure 6A,B) [111]. These nanoparticles can pass the blood–brain barrier and prevent bacterial growth in S. aureus-infected rabbit brains. One recent study showed that hydroponically modified CAPs and rifampicin synergistically treated multi-drug resistant and non-resistant TB and delayed rifampicin resistance [113]. Thus, CAP nanostructures that encapsulate and distribute antibiotics may improve the therapeutic effectiveness of combination therapies.

The advantages of the synthetic polymer analogs of CAPs include lower cost and improved enzymatic stability [114]. Comparable antibacterial processes can be found in quaternary ammonium and phosphonium polymers, which mimic CAPs. Figure 6C,D show the self-assembly of micellar nanoparticles that are made from a CAP-mimicking, amphiphilic triblock polymer. These nanoparticles suppress Gram-positive bacteria, MRSA, and fungi by destroying their membranes, and they do so without causing hemolysis at any dose. Even against Gram-negative *E. coli* and Gram-positive *S. aureus*, CAP-mimicking poly[2-(tert-butylamino)ethyl methacrylate] nanofibers containing Ag nanoparticles showed promising results [115].

Chitosan: Besides synthetic polymers, chitosan, which is a natural cationic polysaccharide polymer, exhibits antibacterial properties. Polycationic chitosan, and its derivatives, are antibacterial, due to their polycationic properties. The electrostatic contact increases the microbial wall permeability, and chelating essential trace metals inhibits enzymes [116]. Due to its larger surface-area-to-volume ratio and microbe attraction, nanoscale chitosan is a better antibacterial treatment than chitosan solution [117]. Chitosan nanoparticles had a MIC of 0.25 g/mL against *E. coli* and *S. aureus*, compared to 20 g/mL for normal chitosan molecules. Chitosan nanoparticles kill fungi and Gram-positive bacteria more effectively than Gram-negative bacteria [118]. In addition, Friedman and colleagues found that nanoparticles that are made of chitosan and alginate have direct bactericidal and anti-inflammatory capabilities by reducing P. acnes-induced cytokine production [119]. These nanoparticles proved to be a promising topical dermatologic therapy when they were encapsulated with benzoyl peroxide, which is an acne medication. Chitosan is hydrophilic and polycationic, making it a good carrier for antibiotics or a coating biomaterial for stabilizing metallic nanoparticles [120].

### 4.2. Drug Delivery

Nanoparticles provide antibacterial agents, which is another important use. Nanoparticle-based medication delivery might overcome antibiotic systemic toxicity, drug uptake and efflux, biofilm development, and intracellular bacterial infection. Surface modification with targeting ligands or microenvironment responsiveness can focus the nanoparticles on the infection site, improving therapeutic efficacy and reducing antimicrobial medication adverse effects. The nanoparticle distribution of antimicrobial medications also improves the hydrophobic drug solubility, the systemic circulation time, the drug half-life, and the drug release, which may minimize systemic adverse effects and the administration frequency [84,123,124,125,126]. Liposomes, solid lipid nanoparticles, polymers, silica, and Au nanoparticles have been produced to perform this [84,127]. Abelcet, AmBisome, Amphoteric, and Fungisome are some of the liposomal/lipid complex antibiotic delivery technologies that are licensed for human use.

Antibiotic resistance prevents microbial cells from attaining harmful drug concentrations through reduced uptake and enhanced efflux [77]. The outer membrane of Gram-negative bacteria, such as *P. aeruginosa* and *E. coli*, may also reduce the uptake of hydrophobic antibiotics, such as beta-lactams and macrolides [128]. An overexpression of transmembrane pumps increases efflux and confers MDR on microorganisms, typically resulting in resistance to chloramphenicol, fluoroquinolones, and macrolides [128]. Two recent reviews [129,130] have found that some nanoparticle delivery vehicles weaken these resistance mechanisms. Fusogenic liposomes that are made of specific lipids can fuse quickly with microbial plasma membranes and deliver a high drug concentration into the cytoplasm, saturating the transmembrane pumps [131].

Nanoparticle-based antibiotic delivery may help to fight biofilms and intracellular bacteria, which cause persistent infections that are hard to treat with traditional antimicrobials. Liposomes and lipid-/polymer-based nanoparticles shield antibiotics from enzymes and promote penetration, boosting their efficiency against biofilm-forming bacteria [132]. Liposome biofilm adherence might be increased by lipids such as phosphatidylinositol and stearyl amine [133]. Nanoparticles infiltrate the host cells through endocytic/phagocytic pathways and release their antibiotic payload into infection sites, due to their tiny size. The mononuclear phagocyte system clears nanoparticles from the body and houses numerous intracellular microorganisms [134]. Polyethylenimine-coated mesoporous silica nanoparticles that were loaded with rifampin were more effective against Mycobacterium tuberculosis-infected macrophages than free rifampin [135]. Nanoparticles for anti-biofilm and intracellular infection therapy have been widely examined in recent reviews [136].

Conjugating several antibiotic copies on nanomaterial surfaces can boost antibacterial effectiveness because some antibiotics interact with bacterial surface components. Au nanoparticles can attach antibiotic medicines to a solid surface and boost their antibacterial activity by interacting with the cell walls [137]. The effectiveness of vancomycin-capped Au nanoparticles in killing vancomycin-resistant Enterococcus and *E. coli* was increased by a factor of 64 compared to vancomycin alone [138]. Several studies have shown that the antibacterial activity of inert nanoparticles can be enhanced by the introduction of chemicals that are either inactive as antibiotics or are less active than antibiotics. Amino-substituted pyrimidine, which is inactive on its own, demonstrated a significant antibacterial effect against MDR clinical isolates after being conjugated on a Au nanoparticle surface [139].

Nanoparticles also deliver nitric oxide (NO), which is a short-lived gaseous antibacterial agent. NO inhibits DNA replication, cell respiration, and reactive nitrogen intermediate production, which makes it antibacterial [140]. These pathways prevent bacterial resistance to exogenous NO treatments [141]. Review publications [142,143] address several nanoparticle platforms for NO delivery. For instance, silica nanoparticles that are produced with NO donors (e.g., diazeniumdiolate) have outstanding antibacterial and biofilm-preventing activity (>99.9%) against *P. aeruginosa* and *E. coli* [144]. When NO donors were encapsulated in biomaterials, such as PAMAM dendrimer and chitosan, these nanoparticles had even greater bactericidal and anti-biofilm characteristics [145]. Besides encapsulating NO-donating compounds, Friedman and colleagues created a sol–gel-based nanoparticle system that can transport gaseous NO from the thermal reduction of nitrite and release it in a regulated and sustained way [146]. NO nanoparticles inhibited several bacteria, even drug-resistant ones. This solution holds NO in a stable state when it is dry and releases gaseous NO when it is wet, making it promising for the topical treatment of wounds and afflicted regions [147]. An MRSA-infected murine wound model showed rapid wound healing and reduced bacterial burden [148].

A combination antibiotic treatment may prevent and treat drug resistance [149]. Additive or synergistic effects can boost medication potency and antibacterial activity. Resistance to various drugs with diverse modes of action requires numerous gene changes in the same bacterial cell, which is unlikely. Nanoparticles might deliver several antibiotics and antimicrobial nanomaterials without synergistic/additive off-target effects. Rifampin and azithromycin-loaded PLGA nanoparticles were more effective against chlamydial infections than either treatment alone [150]. Mesoporous silica that was loaded with peracetic acid and Ag nanoparticles maintained their release and killed antibiotic-resistant and biofilm-forming *S. aureus* [151].

Antimicrobial drugs could be more effective if they were delivered to the location of infection via tailored nanoparticles rather than random ones. The treatment of slow-growing or dormant bacterial infections, which are notoriously difficult to treat and require regular high doses of antibiotics, may also benefit from this [152]. Ligand-modified nanoparticles are used in conventional targeting because of their specificity for binding to receptors on the surface of bacteria. Chlamydia infections, which upregulate folate receptor expression, were treated with azithromycin and rifampicin that were given by PLGA nanoparticles that were conjugated with folate. Liposomes containing ciprofloxacin that were mannose-conjugated showed high selectivity for alveolar macrophages and successfully cured intracellular respiratory tract infections [153].

**Figure 7:**
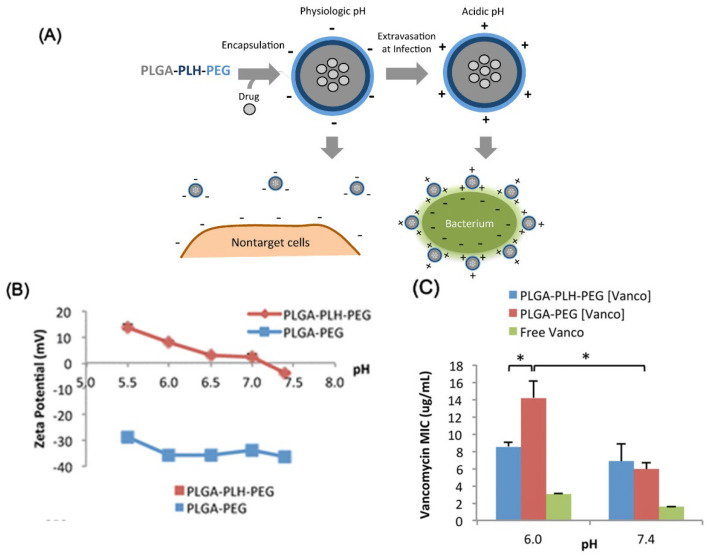
(**A**) Schematic of the nanoparticle-mediated drug targeting bacterial cell walls. A small negative charge and surface PEGylation prevent nanoparticles from attaching to nontarget cells or blood components at physiologic pH 7.4. The surface-charge-switching process activates at weakly acidic infection sites, attaching nanoparticles to negatively charged bacteria. (**B**) PLGA—PLH—PEG nanoparticles convert from anionic to cationic when the pH drops. (**C**) Minimum inhibitory concentrations (MIC) of *S. aureus* vancomycin formulation. * indicates *p* < 0.05. Reproduced with permission from [155]. Copyright American Chemical Society, 2012.

A low pH, enzyme overexpression, localized bacterial toxins, and ligand-targeted nanoparticle delivery are some of the other targeting strategies that have been used [154]. Antibiotic efficiency is reduced due to the local acidity that is caused by the bacterial metabolism and the host immune response [155]. This process is the basis for the discovery of pH-responsive, surface-charge-switching nanoparticles that mask non-specific interactions at pH 7.4 but bind strongly to bacteria at pH 6.0 (Figure 7A,B). Vancomycin that is enclosed in nanoparticles is superior to free drugs at an acidic pH. (Figure 7C). Carboxyl-modified gold nanoparticles can be adsorbed to the exterior phospholipid layer of liposomes, allowing for the liposomes to be turned off at a neutral pH and turned back on at an acidic pH [156]. The combination of Au nanoparticles and liposomes in hydrogel allows for sustained localized drug delivery [157].

The enzymes and toxins that are produced by bacteria can be employed for site-specific applications. By incorporating themselves into the liposome membranes and releasing the encapsulated therapeutic drugs, novel liposomes that were generated by Zhang and colleagues can selectively deliver antibiotics to the areas of bacterial infection [158]. In order to ensure that only bacteria-producing lipase is treated with vancomycin, Wang and coworkers created a lipase-sensitive polymeric nanogel [159]. The drugs are released from the polymeric nanogel when bacterially produced lipase breaks down the nanogel’s polyphosphoester core and poly(e-caprolactone) barrier. The polymeric nanogel, which has been coupled with macrophage-targeting ligands such as mannose, first attaches to macrophages, then accumulates at the bacterial infection sites via macrophage-guided transport, and finally releases the antibiotics upon contact with the lipase-secreting bacteria [160].

## 5. Preclinical and Clinical Translation

### 5.1. Preclinical Translation: Animal-Tested Antimicrobial Nanoparticles

According to the type and the place of infection, nanoparticles that are compatible with the biological environment should be used. In the following subsections, the studies that have evaluated different nanoparticles against infections in animal models are discussed.

#### 5.1.1. Skin and Subcutaneous Region Infection

Bacteria easily settle in skin lesions including atopic dermatitis and chronic wounds, contributing to infection-induced inflammation and disease progression [161]. Due to the obvious skin appearance, skin and subcutaneous infection are the best infection model for animal-based research of nanomedicine’s antimicrobial effectiveness. Topical or subcutaneous bacterium injections establish this infection model easily. Topical, subcutaneous, and intravenous nanoparticle distribution can treat cutaneous and subcutaneous infections. Au nanoparticles that were coated with chitosan and 2-mercapto-1-methylimidazole (MMT) interacted multivalently with bacterial membranes [162]. A gelatin wound dressing was made from nanoparticles and the nanoparticles were applied to a rabbit back wound that was infected with MRSA. The nanocomposite-treated wound closed by 92% after 16 days, while the gauze-treated wound closed by 67%. Liu et al. [163] developed polydopamine-coated Au nanorods for subcutaneous infection chemo-photothermal treatment. The polydopamine-coated nanorods loaded antibacterial Ag efficiently. Fluorescence imaging showed that this platform became positively charged in the acidic abscess, allowing bacteria to accumulate in the infection site. The loaded Ag released the pH sensitively. Under near-infrared (NIR) irradiation, mice received this nanosystem intravenously in order to cure a subcutaneous abscess. NIR hyperthermia increased Ag release and MRSA killing for abscess ablation and wound healing.

Garlic contains antimicrobial allicin [163]. Sharifi-Rad et al. [164] treated MRSA-infected mice with allicin and Ag nanoparticles. The allicin–Ag nanoparticle ointment inhibited the skin MRSA infection synergistically. A photothermal nanocomposite of HA-templated Ag nanoparticles combined with graphene oxide was created to treat skin S. aureus infection [165]. Bacterial hyaluronidase destroyed HA to liberate Ag. NIR light on graphene oxide nanoparticles localized hyperthermia in order to kill the microorganisms. In the in vivo skin wound infection investigation, the nanoparticles with NIR had two orders fewer bacteria than the control and NIR alone. Bacterial consortium and inflammation can result from CVC exposure. Ribeiro et al. [166] immobilized Slavonian A-functionalized SPIONs on CVC for antibacterial prophylaxis. CVC (40 mm) containing 20 μL of 1 × 109 CFU/mL K. of pneumonia caused mice to develop skin infections. A diode laser (808 nm) on the CVC for five minutes reduced the bacterial survival by 88%. The antimicrobial activity lasted for seven days. Cytokines lowered the inflammation. Acetylcysteine-coated Prussian blue nanoparticles enabled photothermal treatment [167]. Mucolytic antibacterial acetylcysteine and Prussian blue nanoparticles are NIR-triggered photothermal agents [168]. K4Fe(CN)6 and FeCl3 co-precipitated acetylcysteine-coated nanoparticles and NIR (980 nm) on the nanoparticles at 50 μg/mL killed *S. aureus* and *E. coli* by 74% and 75%, respectively. Subcutaneous abscesses were cured by NIR exposure following nanocomposite injection.

Carvacrol was incorporated into poly(-caprolactone) (PCL) nanocarriers and combined with hydrogel for topical distribution [169]. Monoterpene carvacrol kills several species of bacteria [170]. Bacterial lipase released carvacrol from enzyme-sensitive nanoparticles. Nanoparticle incorporation increased the carvacrol epidermal deposition from 0.04 to 0.96% of the administered dose in the dermatokinetic investigation. Carvacrol-loaded hydrogel nanoparticles reduced the MRSA burden by 99.97% in pig skin burn wounds. The hair follicles held 25% of the skin bacteria [171]. Eliminating hair follicle bacteria is challenging. Hsu et al. [172] created chloramphenicol-loaded lipid-based nanocarriers for follicular MRSA elimination. DMPC, or DA, was added to liposomes in order to create flexible vesicles for easy extrusion into the follicles. Flexible liposomes containing DMPC and DA increased intrafollicular drug uptake by 1.5- and 2-fold, respectively. Liposomes that were used topically for seven days did not cause skin irritation. Lipid-based nanoparticles can also be used to kill MRSA by combining SME and oxacillin in NLCs [173]. Cationic NLCs could disrupt MRSA membranes and leak proteins. Oxacillin entered the cytoplasm after membrane breakdown. Topical NLCs reduced the MRSA burden by four logs in mouse skin abscesses and NLCs restored the skin architecture and the barrier function.

Yang et al. [174] created lipid bilayer-coated gentamicin-loaded MSNs. Ubiquicidin adorned the MSN bilayer shells. Bacterial toxins could quickly release gentamicin from the lipid bilayer. Planktonic and intra-macrophage *S. aureus* showed rapid antibiotic release. Mice received intracellular S. aureus subcutaneously. After two days, the animals received nanocomposite intravenously. After PBS and free-medication injections, the infected regions had 2.3 × 107 and 8.4 × 106 CFU/mL, respectively. The nanoparticles reduced the bacteria to 1.5 × 104 CFU/mL. The surfactants formed micelles. The antibacterial SMEs were cationic surfactants that formed nanoscale micelles [175]. In the mouse model of subcutaneous MRSA abscess, topically administered SME micelles reduced the bacterial load by 1.6 × 104-fold compared to the vehicle control. Micelle’s intervention on healthy mouse skin caused minimal cutaneous irritation, suggesting that it is a safe anti-MRSA therapy.

#### 5.1.2. Pulmonary Infection

Pneumonia, TB, and cystic fibrosis are caused by respiratory tract bacteria. Nanoformulations were administered intravenously or intratracheally to animals with lung infections. Tigecycline was the model antibiotic that was encapsulated in ICAM1-conjugated β-Ga2O3:Cr3+ nanoparticles by Kang et al. [176]. Inflammatory endothelial cells express ICAM1. Bioimaging semiconductor β-Ga_2_O_3_:Cr^3+^ is luminous [177]. In order to create TRKP-infected pneumonia mice, intratracheal tigecycline-resistant K. pneumoniae (TRKP) was injected into the lung. After 12 days, only the intravenous nanoparticle-treated animals survived the pulmonary infection. The free-drug-treated mice at 45 mg/kg had an 83% survival rate, which was lower than the nanocarrier-treated mice at 15 mg/kg. From 5 to 24 h post-injection, the nanoparticle-treated lung showed increased fluorescence intensity, suggesting targeted administration boosted nanoparticle accumulation in the diseased area.

Polymer-based nanocarriers alleviate *P. aeruginosa*-induced lung infection. Inhaled tobramycin cannot permeate DNA-rich lung mucus [178]. Deacon et al. [179] created tobramycin-loaded chitosan/alginate nanoparticles with DNase to reduce mucus viscoelasticity by DNA breakage. Pretreatment with biopolymer nanoparticles before lung infection with *P. aeruginosa* doubled the survival rate from 40% with free antibiotics to 80%. DNase-containing nanoparticles penetrated the cystic fibrosis sputum more effectively. Intratracheal PLGA nanoparticles carrying esculentin-1a cured lung infection in a study by Casciaro et al. [180]. PVA stabilized the nanoparticles. The pulmonary mucus easily permeated the neutral hydrophilic nanoparticles. Esculin-1a-loaded nanocarriers reduced CFU by three logs in *P. aeruginosa*-infected mice. Free esculentin-1a had 17-fold less anti-*P. aeruginosa* action. Micelle nanocarriers were made by conjugating vancomycin with amphiphilic PEG-co-PCL copolymer via pH-cleavable hydrazone linkages [181]. The nanocomposite contained on-demand ciprofloxacin. Under acidic conditions, the nanocomposite’s vancomycin shell opens, disrupting the hydrophilic/lipophilic balance and increasing the micelle size, which helps the lipase that is overexpressed in the infection site to degrade PCL and release ciprofloxacin to kill *P. aeruginosa*. The micelles reduced the lung bacterial load and the alveolar damage in *P. aeruginosa*-infected mice.

A ROS-responsive 4-(hydroxymethyl) phenylboronic acid pinacol ester-modified α-cyclodextrin was coated with phospholipids in order to generate lipid-coated nanoparticles in order to deliver moxifloxacin to infected lung tissue and sustain drug release [182]. In the inflammatory zone, nanocarriers that were coated with 1,2-stearoyl-sn-glycerol-3-phosphoethanolamine (DSPE)-PEG-folic acid allowed sputum to penetrate and target macrophages with overexpressed ROS. Mice with lung *P. aeruginosa* infections received the nanosystem intravenously. Moxifloxacin could increase the survival rate from 20% to 40% following nanoparticulate encapsulation. Nanocomposite therapy eliminated the lung pathogen colonies. PEGylated phosphatidylcholine-rich nanovesicles were tested for infectious pneumonia treatment [183]. Ciprofloxacin-loaded nanovesicles targeted lung surfactants. Intracellular MRSA may then disappear. After an intravenous injection of lipid nanovesicles, lung ciprofloxacin accumulation increased 3.2-fold in vivo. The control medication and nanovesicles reduced the pulmonary MRSA from 4.9 × 108 to 1.2 × 108 and 6.3 × 107 CFU, respectively.

Antimicrobial peptide NZX inhibits drug-resistant M. tuberculosis. Due to the macrophages’ high absorption of MSNs, Tenland et al. [184] tried to entrap NZX in them in order to cure tuberculosis. Nanoparticles killed intra-macrophage bacteria more effectively than free NZX. In the mouse tuberculosis model, intratracheal free peptide and NZX-containing MSNs lowered lung M. tuberculosis CFU by 84% and 88%, respectively. MSNs also actively targeted lung infections [185]. Vancomycin-loaded nanoparticles were coupled with *S. aureus*-recognizing cyclic 9-amino-acid peptide CARGGLKSC (CARG). CARG bound only to S. aureus in vitro. Intravenous CARG-conjugated nanoparticles had eight-fold more lung deposition than non-targeted nanoparticles. *S. aureus* that was instilled intratracheally into mouse lungs caused 67% mortality after 24 h. CARG-conjugated MSNs enhanced the survival rate to 100%. All MSN-treated mice survived for 20 days.

#### 5.1.3. Gastrointestinal (GI) Infection

Oral antimicrobial nanoparticles treat gastrointestinal infections. Nanocarriers protect antibiotics against GI fluid breakdown. Bioadhesive nanoparticles prolong GI tract retention for oral bioavailability. Oral MSNs are suitable for GI medication enzymolysis protection. Zhao et al. [186] created intestine-targeted antimicrobial peptide defensin-loaded MSNs. The stomach degrades defensin. Succinylated casein, which intestinal protease may break down, was coated onto MSNs for intestinal targeting. In acidic conditions, casein ornamentation lowered the defensin release, while trypsin controlled it. Orally gavaged multidrug-resistant *E. coli* caused intestinal illness. Nanoparticles were taken orally daily for five days. The casein-coated nanomedicine reduced the bacteria colonization more than the free ciprofloxacin. Compared to the non-coated MSNs and the free peptides, the casein-coated nanoparticles lowered the intestinal TNF-α 1.5- and 2.2-fold.

Montmorillonite is a smectic clay with mucoadhesive and EPS-attaching properties [187]. H. pylori infection in GI patients was treated with a montmorillonite-cationic PEI metronidazole nanocomposite [188]. By acting as a biomimetic building block, montmorillonite can zero in on bacteria, while PEI can facilitate bacterial membrane rupturing, which improves the entry of antibiotics into the cytoplasm. Nanoparticles that are administered orally showed widespread distribution in the stomach tissue, demonstrating their mucoadhesion. Using nanocarriers to eliminate *H. pylori* in the gastrointestinal tract led to a reduction in gastric ulcers and inflammation. Compared to omeprazole, amoxicillin, and metronidazole, this triple therapy was more effective against germs. In order to create biomimetic nanocarriers for targeting *H. pylori*, the gastric epithelial cell membrane was coated onto PLGA nanoparticles [189]. *H. pylori* was attracted to the biomimetic nanocarriers 10 times more than to the uncoated nanoparticles. After the oral administration of the biomimetic nanoparticles and the free medicine, the bacterial burden in the stomachs of the infected mice was reduced from 1.6 105 CFU/g to 6.5 103 and 5.0 104, respectively.

#### 5.1.4. The Other Infection Sites

Antibacterial nanoparticles have been used to treat systemic, bone, and vaginal infections. Systemic bacterial infections cause bacteremia and sepsis [190]. Rai et al. [191] coupled high-density antibacterial peptides on Ag nanoparticles in order to eliminate MRSA. This study used cecropin–melittin. Nanoparticles that are 14 nm might be regulated. Bacteremia was treated in septic-like animals with intraperitoneal Au nanoparticles. The circulation the MRSA concentration was two logs lower in the peptide-conjugated nanoparticle group. The spleens received most of the nanoparticles. Metallic nanoparticles were used to treat bone infections.

Ag–Cu nanoparticles by Qadri et al. [192] eliminated *S. aureus* bone infiltration in mice. Boron was added to nanoparticles in order to prolong antibacterial action because its anticorrosive properties delayed Cu oxidation [193]. The nanoparticles measured 27 nm. *S. aureus* was inserted into the mice’s bones with a silk suture in order to cause osteomyelitis. The 1 mg/kg intravenous nanoparticles reduced the bacterial CFU 10-fold compared to the control. The *S. aureus* bone accumulation was also suppressed intramuscularly. Magnetic Fe_3_O_4_ nanoparticles and heat-disrupted biofilm were used to cure osteomyelitis [194]. The S. aureus-infected bone received SPIONs. Infected bone magnetic fields were able to heat the implant to 75 °C. Vancomycin in the femoral canal during heating killed the biofilm microorganisms. Vancomycin and heat had 24% more bone volume than the infection control (18%). ZnO nanoparticles showed a low-concentration of antibacterial activity [195]. A PVA hydrogel containing 10 nm ZnO nanoparticles treated vaginitis vaginally [196]. Vaginal *E. coli* inoculation for five days caused vaginitis in mice. The nanoparticles reduced the CFU in vaginal washes. The histological epithelial exfoliation scores and the *E. coli* counts were consistent.

### 5.2. Clinical Trials

The good news is that nanosystem-based antibiotics, antitoxin compounds, and antimicrobial peptides have been transferred to the clinic after substantial research into revolutionary antimicrobial delivery systems to combat antibiotic resistance. Many are still undergoing clinical testing (Table 1).

In a Phase 1 trial in healthy volunteers, Lipoquin was used to inhale ciprofloxacin-loaded liposomes [196]. In 21 adult CF patients, a Phase 2a multi-center 14-day trial assessed Lipoquin’s efficacy, early safety, and pharmacokinetics. In similar regions, ORBIT-3 and ORBIT-4 were international, double-blind, randomized, Phase 3 trials of inhaled liposomal ciprofloxacin’s safety and efficacy [197,198]. Amikacin-loaded liposomes were also studied clinically. Individuals with a treatment-refractory nontuberculous mycobacteria lung infection on a stable multidrug regimen were compared to a placebo over the course of 84 days in a double-blind, randomized study testing the efficacy, safety, and tolerability of a once-daily amikacin 590 mg treatment [199]. For 18 months, patients with cystic fibrosis who had chronic Pseudomonas aeruginosa infections in Phase 2 trial breathed in 560 mg of amikacin-loaded liposomes once per day [200]. Liposomal amikacin (590 mg once per day for 12 months), in combination with the current gold-standard mycobacterial multi-drug regimen, for the treatment of mycobacterium abscesses in pulmonary illness will be tested in a Phase 2 trial in order to determine its efficacy, safety, and tolerability [201]. Studying the long-term safety and acceptability of inhaled amikacin-loaded liposome (590 mg/day) in individuals with cystic fibrosis and persistent Pseudomonas aeruginosa infection will be carried out in a Phase 3 clinical investigation [202].

Antibacterial drugs may benefit from a nano-preparation that targets bacterial toxins. In 2016, the first human monoclonal antibody targeting Clostridium difficile toxin B was approved, which was bezlotoxumab [203]. Monoclonal antibodies targeting *S. aureus*’ α-toxin and *P. aeruginosa’s* type III toxins secretory moiety are in clinical trials [204]. A broad-spectrum antitoxin liposomal compound (CAL02) has synergistic effects with medicines or antibiotics and can save mice from serious infections, such as staphylococci, by adsorbing toxins [205].

Antimicrobial peptides have broad-spectrum antibacterial action and little resistance risk due to their fast death [206]. Antimicrobial peptides target bacterial cell membranes. Nisin, nucleic acid, RNA, protein, and statins are intracellular targets [206].

**Table 1:** Nanomaterial-based antimicrobials in different stages of the clinical trial.

Antimicrobial	Trial Phase	**Application**	Ref.
Abelcet	Marketed	Fungal infection	[207]
AmBisome	Marketed	Fungal infection	[208]
Amphotec	Marketed	Fungal infection	[209]
Fungisome	Marketed	Fungal infection	[210]
Ciprofloxacin	Phase 1	*Pseudomonas aeruginosa*	[211]
Ciprofloxacin	Phase 2a	*Pseudomonas aeruginosa*	[211]
Ciprofloxacin	Phase 3	Bronchiectasis and Chronic *P. Aeruginosa* Infection	[197]
Ciprofloxacin	Phase 3	Non-cystic fibrosis bronchiectasis (NCFB)	[212]
Amikacin	Phase 2	Mycobacterium Infections, Nontuberculous	[199]
Amikacin	Phase 3	Cystic Fibrosis Patients with Chronic Pseudomonas aeruginosa Infection	[202]
Amikacin	Phase 2	Mycobacterium Infections, Nontuberculous Mycobacteria, Atypical	[201]
Amikacin	Phase 3	Mycobacterium Infections, Nontuberculous	[213]
Amikacin	Phase 2	Cystic Fibrosis	[200]
Biological: CAL02	Phase 3	Severe community-acquired pneumonia	[205]
Biological: GS-CDA1 Biological: MDX-1388	Phase 2	Clostridium Difficile Associated Disease	[214]
Novacta biosystems (NVB-302)	Phase 1	Clostridium difficile	[215]
Human lactoferrin (hlf1-11)	Phase 2	Infection following transplantation	[216]
(a potent cyclic lipodepsipeptides antibiotic) Wap-8294A2	Phase 2	Gm+ve bacteria (VRE and MRSA)	[217]
The specifically targeted antimicrobialpeptide (C16G2)	Phase 2	*Streptococcus* mutans	[218]
Antimicrobial Peptide (DPK-060)	Phase 2	Acute external otitis	[219]
LTX-109 (Lytixar)	Phase 2	Nasal decolonization of MRSA Impetigo	[220]
p2TA (AB 103)	Phase 3	Necrotizing soft tissue infections	[198]
Surotomycin	Phase 3	Clostridium difficile	[221]
Ramoplanin (NTI-851)	Phase 2	Clostridium difficile	[222]

## 6. Concluding Remarks

Nanotechnology is promising for microbial illness treatment. Due to its high adjustability and broad range of adaptation, antibiotics with nanomaterials are a more cost-effective option for macrophage persister cells and biofilm infections. Nano-antibiotic systems can target, penetrate, absorb, and change infectious microenvironments, and combine with other treatment techniques due to their nanomaterial design. Thus, nanomaterials have considerable potential to improve antibiotic efficacy. Clinical translation must first resolve various issues and testify carefully about in vivo toxicity and clinical effects. Nano-antibiotics for resistant bacterial infections will require long-term research and practice before their widespread use. Nanomaterials are still a promising antibiotic-resistance-fighting option. We think that nano-antibiotics can combat bacterial resistance and save more lives soon.

## Data Availability

The study did not report any data.
